# The Remote Completion Rate of Electronic Patient-Reported Outcome Forms Before Scheduled Clinic Visits—A Proof-of-Concept Study Using Patient-Reported Outcome Measurement Information System Computer Adaptive Test Questionnaires

**DOI:** 10.5435/JAAOSGlobal-D-19-00038

**Published:** 2019-10-02

**Authors:** Peter A. Borowsky, Omar M. Kadri, Jason E. Meldau, Jacob Blanchett, Eric C. Makhni

## Abstract

**Introduction:**

Patient-Reported Outcome Measurement Information System (PROMIS) questionnaires are amenable to remote administration. This study sought to determine remote completion rates of PROMIS questionnaires before clinic visits.

**Methods:**

Patients were e-mailed a set of PROMIS forms. Completion rates were analyzed by visit type, provider seen, and patient demographics.

**Results:**

Seven hundred forty total appointments were included. Sixty-seven percent of encounters had previsit form completion. High completion rates were found for all visit types (74%, 67%, and 64% for new, return, and postoperative visits, respectively). Women had a higher completion rate than men (71% versus 64%; *P* = 0.031). White patients (72%; *P* = 0.001) and patients in the third median household income quartile ($53,725 to $83,088; 72%; *P* = 0.008) had higher completion rates than their respective counterparts.

**Conclusion:**

Most patients remotely completed PROMIS forms. The efficiency and accessibility of PROMIS forms may help improve ease of collection of patient-reported outcomes.

Despite increased emphasis and incentives for collecting and reporting patient-reported outcome (PRO) measures in orthopaedics, the administration of these measures continues to be a burdensome task.^1,2,3,4,5^ Costs, survey fatigue leading to unreliable results, and interruptions to clinic workflow are obstacles for widespread PRO collection.^6,7,8,9^ Recent studies have shown that use of the National Institutes of Health Patient-Reported Outcome Measurement Information System (PROMIS) forms, and in particular the Computer Adaptive Test (CAT) version, greatly reduces these burdens, while remaining as effective and reliable as traditional PRO measures.^10,11,12,13,14^

As health care transitions to a value-based reimbursement platform, there is an increasing need to collect clinical outcomes and PROs on a routine basis. Such practices help providers and policy makers determine the value of different clinical interventions. Unfortunately, collection of outcomes from postoperative patients has proven to be particularly challenging, with patient engagement dropping as time elapses after surgery.^15^

Despite the efficiency and accessibility of PROMIS CAT forms, no study has investigated whether these forms can be successfully administered to patients before their clinic visits. Successful previsit completion would decrease the logistic and administrative burdens of coordinating outcome collection in the busy ambulatory setting. Therefore, the purpose of this study was to determine the rates of completion of PROMIS CAT forms in a cohort of patients who received these forms electronically before their scheduled clinic visits. A secondary goal was to analyze the relationship and association of patient-centric factors with remote completion rates of PROMIS CAT forms. We hypothesize that, given the nature of PROMIS CAT forms, most patients would successfully complete their PRO forms before their clinic visit.

## Methods

After Institutional Review Board approval at our institution, all elective, sports medicine patients presenting to the clinics of three fellowship-trained providers (two sports medicine orthopaedic surgeons and one shoulder and elbow surgeon), and who had active e-mail addresses listed in the electronic medical record (EMR), were included in this study. At our institution, EPIC is the EMR system in use. Only patients who were scheduled at least seven days in advance of their appointment were included because PROMIS CAT forms were e-mailed 1 week before the clinic visit. Recruitment was performed at a multisite, integrated healthcare system in a large metropolitan city between December 2017 and February 2018. E-mails with instructions on PROMIS CAT completion were sent 7 days before the scheduled clinic visits. Exclusion criteria included unavailable/inactive e-mail address, appointments scheduled less than seven days prior, no-shows, and visits that were for regularly scheduled imaging review or injections performed in a series (because no change was observed in clinical history or symptoms).

Patients completed an intake form documenting the physician seen and the site of injury or pain (shoulder, elbow, hand/wrist, neck, spine/back, hip/pelvis, knee, and foot/ankle). Forms were completed using a secure, electronic PRO collection platform (REDCap, Vanderbilt University) on a tablet computer (iPad, Apple). Each patient then completed a PROMIS Pain Interference (PROMIS-PI) CAT, a PROMIS Depression CAT, and a PROMIS Physical Function CAT. Patients with upper extremity concerns completed the PROMIS Upper Extremity Physical Function CAT, whereas patients with lower extremity concerns completed the PROMIS Physical Function CAT. Patients completed both physical function forms if they selected both upper and lower extremity concerns in the intake form.

Both the intake form and the questionnaire set were e-mailed to patients before their clinic visits. A standardized e-mail that contained a link to the questionnaires was sent to patients seven days before their visits. Patients who did not complete the forms within the first five days were sent a reminder e-mail 2 days before their visits. All e-mails were sent by the same research assistant using a standardized script and included a statement assuring patient confidentiality and security of information collected. Also included in the e-mails was a statement acknowledging that participation was voluntary and that the questionnaires could be completed during the appointment if patients preferred that option.

The primary outcome was completion of the questionnaire set remotely before arrival for the scheduled appointment. A PROMIS questionnaire set was recorded as completed only if all PROMIS forms were finished before the appointment. For each patient encounter, the type of visit was documented. New clinic visits were categorized as those in which the patient had not previously attended an appointment with any of the three scheduled providers. Return clinic visits were those in which the patient had previously attended an appointment with at least one of the three providers. Postoperative clinic visits were those in which the patient returned to clinic within 90 days after a previous surgery performed by one of the three providers. If more than 90 days had passed since the date of surgery, visits were categorized as return clinic visits. Subgroup analysis for remote completion rates was conducted by patient demographic information (age, sex, and race), body mass index (BMI), tobacco use, and estimated median household income (MHI). Tobacco use included both smoking and smokeless tobacco, with patients' tobacco history recorded as unknown, never, former, or current. Using previously published methodology, a patient's zip code of residence was used to estimate MHI.^16^ This information was obtained from publicly available records (https://factfinder.census.gov/faces/nav/jsf/pages/community_facts.xhtml?src=bkmk).

For the purpose of our study, it was possible that a single patient could be included multiple times in the process of data analysis. For example, if a patient presented during the study period for both a new clinic visit and a postoperative clinic visit, then the patient was assessed for remote PROMIS CAT completion two separate times.

An independent *t*-test was performed to determine whether age was associated with a higher remote survey completion rate. Chi-square analysis was performed to determine whether remote completion rates were associated with patient sex, race, and tobacco use. Analysis of variance tests were used to assess the association of completion rates with both MHI and BMI. SPSS software (Version 22, IBM Inc) was used for all statistical analyses, with *P* values lower than 0.05 considered to be statistically significant.

## Results

**Figure 1:**
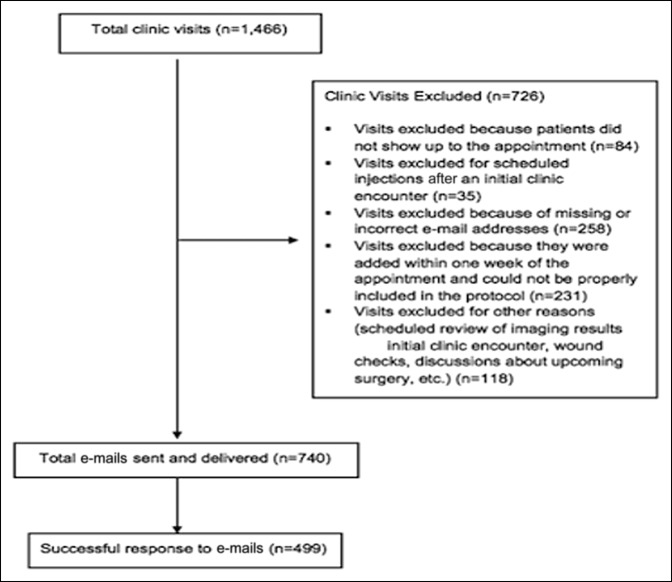
Chart showing inclusion and exclusion criteria for all patients presenting to scheduled clinic visits.

In total, 627 patients, accounting for 740 clinic visits, met the inclusion criteria and were included for the review. Two hundred eighty-five visits were excluded because the patients did not have an active e-mail address listed in their EMR (Figure 1). The average age of the cohort was 50.5 years (range 12 to 90 years, SD 17.7). Demographic information of the study participants, both those who completed the forms remotely and those who did not, is presented in Table 1.

**Table 1:**
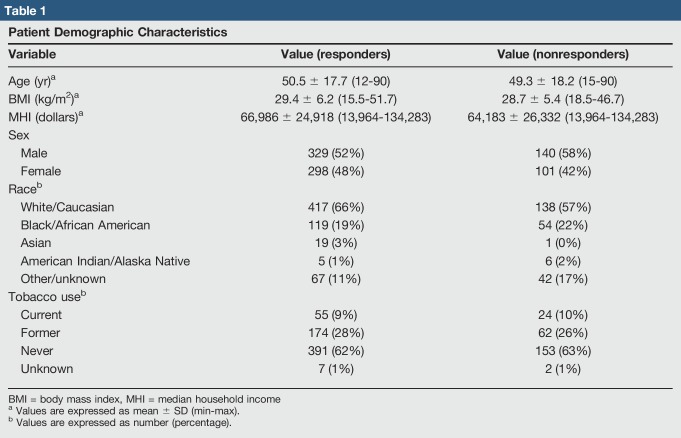
Patient Demographic Characteristics

Variable	Value (responders)	Value (nonresponders)
Age (yr)^a^	50.5 ± 17.7 (12-90)	49.3 ± 18.2 (15-90)
BMI (kg/m^2^)^a^	29.4 ± 6.2 (15.5-51.7)	28.7 ± 5.4 (18.5-46.7)
MHI (dollars)^a^	66,986 ± 24,918 (13,964-134,283)	64,183 ± 26,332 (13,964-134,283)
Sex		
Male	329 (52%)	140 (58%)
Female	298 (48%)	101 (42%)
Race^b^		
White/Caucasian	417 (66%)	138 (57%)
Black/African American	119 (19%)	54 (22%)
Asian	19 (3%)	1 (0%)
American Indian/Alaska Native	5 (1%)	6 (2%)
Other/unknown	67 (11%)	42 (17%)
Tobacco use^b^		
Current	55 (9%)	24 (10%)
Former	174 (28%)	62 (26%)
Never	391 (62%)	153 (63%)
Unknown	7 (1%)	2 (1%)

BMI = body mass index, MHI = median household income

a Values are expressed as mean ± SD (min-max).

b Values are expressed as number (percentage).

**Figure 2:**
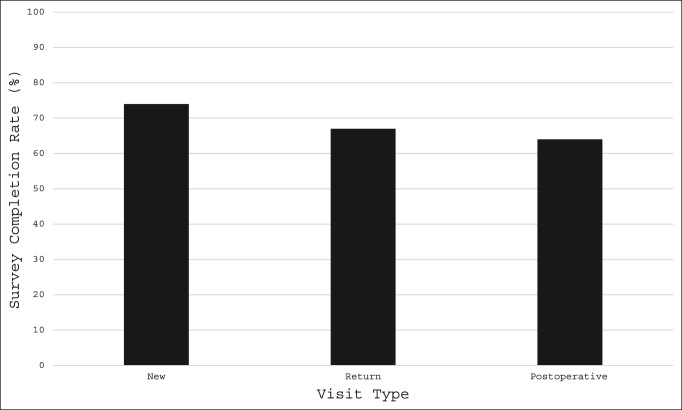
Chart showing remote completion of PROMIS CAT forms by visit type. CAT = Computer Adaptive Test, PROMIS = Patient-Reported Outcome Measurement Information System

The overall survey completion rate was 67% (Table 2). Completion rates did not differ significantly by visit type (*P* = 0.12; range 64% to 74%) or provider seen (*P* = 0.21; range 65 to 70%). We did note, however, that patients had high survey completion rates among all three visit types and all three providers. New, return, and postoperative visits had remote survey completion rates of 74%, 67%, and 64%, respectively (Figure 2). Patients seeing providers 1 and 2 (both sports medicine specialists) had completion rates of 70% and 65%, respectively, and patients seeing provider 3 (a shoulder and elbow specialist) had a completion rate of 68% (Table 2).

**Table 2:**
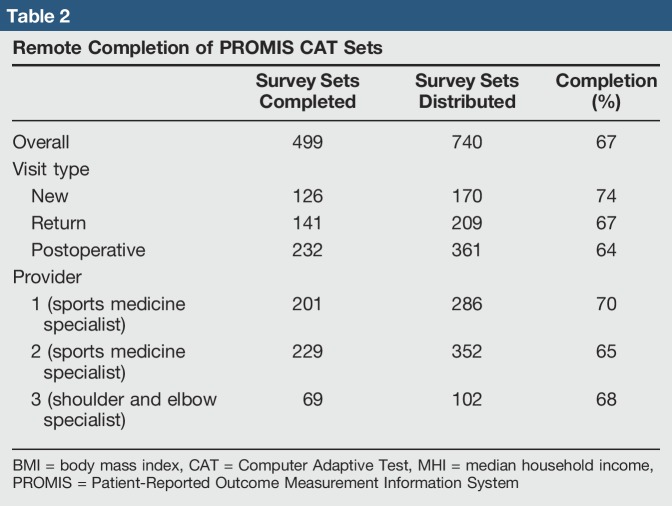
Remote Completion of PROMIS CAT Sets

	Survey Sets Completed	Survey Sets Distributed	Completion (%)
Overall	499	740	67
Visit type			
New	126	170	74
Return	141	209	67
Postoperative	232	361	64
Provider			
1 (sports medicine specialist)	201	286	70
2 (sports medicine specialist)	229	352	65
3 (shoulder and elbow specialist)	69	102	68

BMI = body mass index, CAT = Computer Adaptive Test, MHI = median household income, PROMIS = Patient-Reported Outcome Measurement Information System

**Figure 3:**
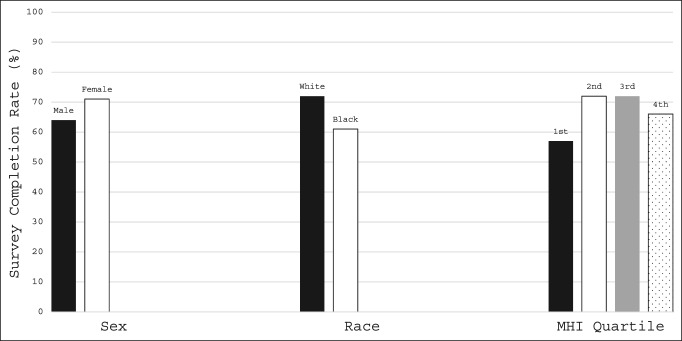
Chart showing patient demographic factors demonstrating differences in survey completion rates. MHI = median household income

**Figure 4:**
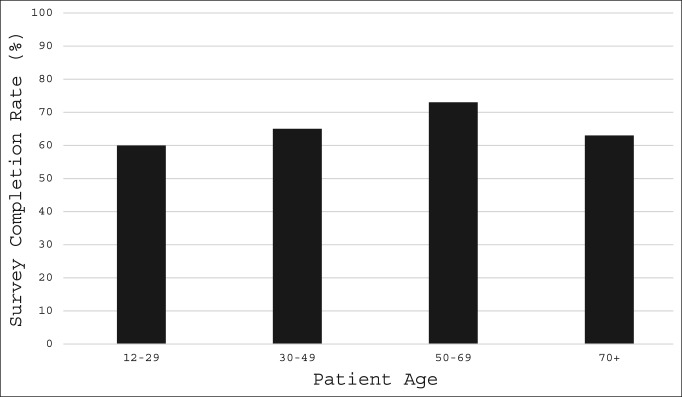
Chart showing remote completion of PROMIS CAT forms by patient age.

Female patients had a significantly higher completion rate for remote PROMIS CAT questionnaire completion than did male patients (71% versus 64%; *P* = 0.031). Completion rates also differed by patient race (*P* = 0.001; range 50% to 83%), with white patients having a higher completion rate than black patients (72% versus 61%; *P* = 0.011). Completion rates also differed by estimated MHI quartile (*P* = 0.008; range 57% to 72%), with patients in the lowest income quartile (<$46,496) having lower completion rates than patients in the second ($46,497 to $53,724; *P* = 0.007) and third ($53,725 to $83,088; *P* = 0.002) income quartiles (Figure 3). No significant differences in remote PROMIS CAT survey completion rates were found between any additional race or MHI quartile patient groups (*P* > 0.05). Remote completion rates did not differ regarding tobacco use (χ^2^ = 2.083; *P* = 0.555; range 61% to 71%), patient age (χ^2^ = 2.645, *P* = 0.45; range 60% to 73%), or patient BMI (χ^2^ = 2.392; *P* = 0.664; range 65% to 83%). We did find, however, that patients aged 50 years or older had higher remote survey completion rates than their younger counterparts (Figure 4). Remote PROMIS CAT survey completion rates stratified by patient demographic factors are presented in Table 3.

**Table 3:**
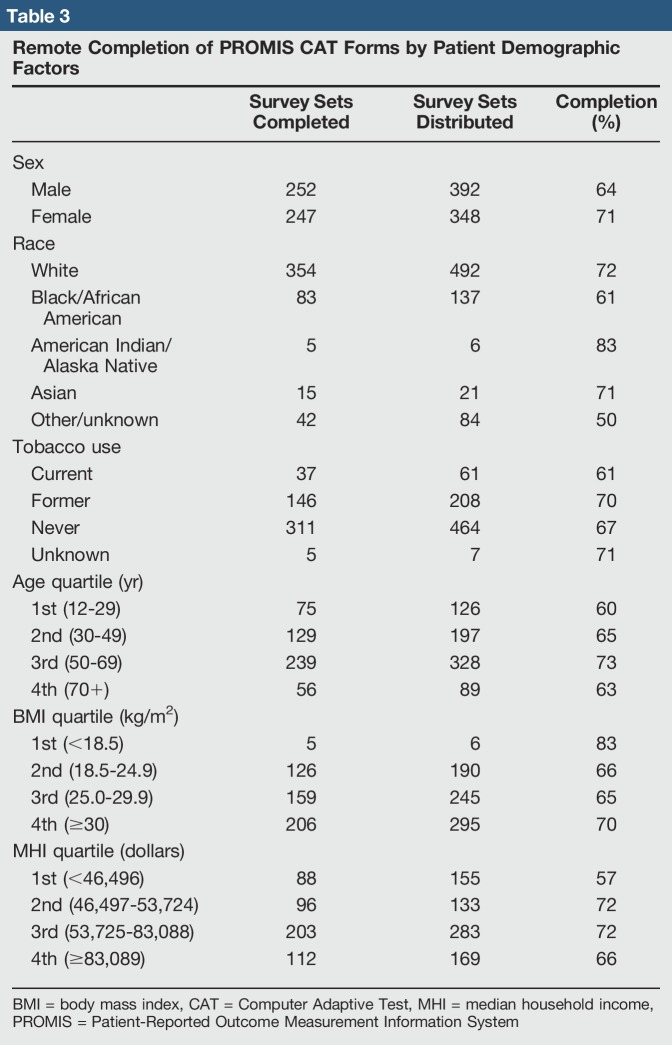
Remote Completion of PROMIS CAT Forms by Patient Demographic Factors

	Survey Sets Completed	Survey Sets Distributed	Completion (%)
Sex			
Male	252	392	64
Female	247	348	71
Race			
White	354	492	72
Black/African American	83	137	61
American Indian/Alaska Native	5	6	83
Asian	15	21	71
Other/unknown	42	84	50
Tobacco use			
Current	37	61	61
Former	146	208	70
Never	311	464	67
Unknown	5	7	71
Age quartile (yr)			
1st (12-29)	75	126	60
2nd (30-49)	129	197	65
3rd (50-69)	239	328	73
4th (70+)	56	89	63
BMI quartile (kg/m^2^)			
1st (<18.5)	5	6	83
2nd (18.5-24.9)	126	190	66
3rd (25.0-29.9)	159	245	65
4th (≥30)	206	295	70
MHI quartile (dollars)			
1st (<46,496)	88	155	57
2nd (46,497-53,724)	96	133	72
3rd (53,725-83,088)	203	283	72
4th (≥83,089)	112	169	66

BMI = body mass index, CAT = Computer Adaptive Test, MHI = median household income, PROMIS = Patient-Reported Outcome Measurement Information System

## Discussion

This study demonstrates that most patients with active e-mail addresses are likely to participate in and complete online, remotely administered PROMIS CAT forms, regardless of visit type (new, return, or postoperative). In particular, both return and postoperative patients had remote survey completion rates equal to or close to the cumulative completion rate for all patients included in this study. Patients who were white, female, and from higher MHI also demonstrated a higher likelihood of remote participation.

Previous studies have reported low success rates with remote PRO administration.^13,17^ In particular, previous researchers have found remote completion rates of only 24% for the Disabilities of the Arm, Shoulder, and Hand (QuickDASH) questionnaire,^18^ the short version of the Patient Health Questionnaire-2,^18^ and the Pain Self-Efficacy Questionnaire^18^ forms; 36% for the Knee Injury and Osteoarthritis Outcome Score^19^ questionnaire forms; and 34% for the health-related quality of life questionnaire forms containing the health-related quality of life tool EQ-5D.^14^ The results from our study demonstrated higher rates of remote completion when using PROMIS CAT forms, likely because of the decreased time to completion of these forms.^20^ Schønnemann et al^19^ found an average time to completion of 11 minutes for QuickDASH questionnaires, which was well above the documented time to completion using other PRO forms. Furthermore, Kortlever et al^14^ found that PROMIS-PI forms required less than one half the amount of time to complete compared with Pain Self-Efficacy Questionnaire forms (30 versus 78 seconds). Many studies have analyzed whether this shorter time to completion leads to inaccurate results; however, PROMIS CAT forms retain the same degree of reliability and exhibit less floor and ceiling effects compared with traditional “legacy” PRO forms.^14^ Our results show that remote completion of PROMIS CAT forms is a feasible way of collecting PROs from patients presenting to elective sports medicine orthopaedic clinics. This is important because, in our clinic in particular, the primary challenges to in-clinic PROMIS CAT completion are the requirement of dedicated research personnel, the continuous yearly training of research personnel and office staff, and interruptions to clinic workflow. Successful remote completion of PROMIS CAT forms before scheduled clinic visits has the ability to minimize the impact of these challenges through reduction of the number of CAT forms that need to be distributed in the clinic setting. In addition, successful remote completion has the ability to increase the cumulative overall rate of PRO completion. Despite these advantages, additional work must be performed to increase remote completion of PROMIS forms by clinic patients.

Although age did not significantly affect the completion rates in our study, patients aged 50 years and older had higher PRO completion rates when compared with their younger counterparts, a finding that echoes previous reports on survey completion rates. In a study performed to assess factors associated with nonresponse to survey studies in the field of orthopaedic surgery, Bot et al^21^ found that younger age was a significant predictor of higher nonresponse rates. Likewise, after analyzing response data from the “45 and Up Study,” a public health, longitudinal campaign in Australia designed to track health outcomes and lead to better understanding of the human aging process, Wang et al^22^ found that individuals aged 55 to 74 years had the highest survey response rates, whereas individuals of younger age were more likely to have higher nonresponse rates. These findings align with our observed results and further suggest a relationship between patient age and the likelihood of remote PRO completion. However, despite these findings, most of our youngest quartile of patients—60%—still completed these PROMIS CAT forms remotely.

Our study did report an impact of MHI on the likelihood of remote PROMIS CAT completion. The relationship between income and access to technology and eHealth literacy (the ability to find, understand, appraise, and apply health information from online sources) has been well established. In a survey analysis of household income and Internet use conducted by the Pew Research Center's Internet and American Life Project, 80% of individuals with household incomes $30,000 to $49,000 use the Internet, compared with over 95% of individuals with incomes over $75,000.^23^ Furthermore, in a systematic review of eHealth literacy by Chesser et al,^24^ the authors found low income to be a common predictor of low eHealth literacy, a finding that may be attributed in part because of their reduced access to technologies necessary to complete PROMIS CAT surveys. Our results support these findings and suggest low income as a possible barrier to the success of remote e-mail administration and completion of PRO. MHI predicts access to and use of resources needed to successfully respond to e-mailed forms and thus may have an impact on the observed completion rates. Therefore, remote administration of PROMIS forms should be used as an adjunct, rather than replacing in-office options.

This study did have important limitations. One such limitation was that patients' e-mail addresses were retrieved from the EMR. Therefore, there might have been patients with valid e-mail addresses that were not included in the medical records. However, because our health system is an integrated, multicenter, and multispecialty system, numerous opportunities exist for patients to update their e-mail addresses in their health records, thereby minimizing this discrepancy. In fact, at every clinic encounter system-wide, this contact information is updated at check-in. Second, by nature of the study design, a selection bias existed for patients with valid e-mail addresses. This might have introduced a selection bias for patients who were younger and had better electronic/online access (e.g. higher socioeconomic status). However, a wide range of MHI and patient age were included in our study population, thereby indicating a diverse group of patients regarding socioeconomic status. MHI as a measure of diversity and inclusion has been used previously in the previous literature.^16^ Given that we selected patients with valid and active e-mail addresses, by nature of the study design, we were unable to collect PROs from patients without active e-mail addresses. However, during our study period, only 285 of the total 1466 clinic visits (19%) were excluded because of the lack of a valid e-mail address. This number is a small percentage of the total population and indicates that remote administration by the use of e-mail may be a valid option for distributing and collecting PROs. A downside of our study design is that we did not track or record information about patients who were excluded from participation. It is possible that the demographics of patients included could differ from the demographics of patients excluded; thus, potentially leading to selection bias. Further studies should address this limitation in the study design.

In addition, all PROMIS forms administered to patients in our study were filled in the English language. Offering additional languages might have improved the completion rates. Furthermore, our study did not use pediatric-specific PROMIS forms for the pediatric patient population. Follow-up studies using pediatric-specific PROMIS measures are needed to better determine remote completion rates of PROMIS CAT forms in the pediatric population.

Another limitation to our study is the inherent presence of selection bias in the elective sports medicine population. Existing literature shows that patients presenting to sports medicine clinics tend to, on average, be younger than patients presenting to clinics of other specialties. In addition, the elective sports medicine population tends to include higher proportions of male patients and patients with lower BMI compared with other patient populations.^25,26^ Given this inherent bias present in the elective sports medicine population, the results of our study cannot be generalized to the success of remote PRO administration and completion in other fields of medicine. In addition, our results cannot be generalized to the association of patient demographics with remote PROMIS CAT completion rates in other patient populations.

## Conclusion

Most patients are willing to complete online-administered PROMIS CAT forms before visits with their orthopaedic care provider, regardless of being scheduled for a new, return, or postoperative visit. Improved access to e-mail or other electronic communication methods may help providers collect outcomes in this efficient manner, thereby reducing the logistic and financial burden of PRO collection in the busy ambulatory setting.
